# A unified multi-modal latent diffusion framework with modality-dropout training

**DOI:** 10.3389/frai.2026.1867917

**Published:** 2026-09-15

**Authors:** S. Remya, Manu J. Pillai, Laveena Herman, B. Niranjan, Somula Ramasubbareddy, Yong Yun Cho

**Affiliations:** 1 Department of CSE, Amrita School of Computing, Amrita Vishwa Vidyapeetham, Amritapuri, Kollam, Kerala, India; 2 Department of CSE, TKM College of Engineering, Kollam, Kerala, India; 3 Symbiosis Institute of Technology, Hyderabad Campus, Symbiosis International (Deemed University), Pune, India; 4 Department of Information and Communication Engineering, Sunchon National University, Suncheon, Jeollanam-do, Republic of Korea

**Keywords:** adaptive guidance fusion controllable image synthesis, CIFAR-10, CLIP, Latent diffusion models, modality dropout, multi-modal conditioning, U-Net

## Abstract

**Introduction:**

Multi-modal conditioning in latent diffusion models—combining text, structural, and spatial guidance signals—substantially improves controllable image synthesis, yet two limitations persist across most existing frameworks. First, conditioning modalities are fused using fixed architectural weights that remain constant regardless of the input, preventing any input-adaptive rebalancing of guidance. Second, most existing multi-modal approaches are designed under the assumption that all conditioning signals will be present at inference time, leaving the model without any way to degrade gracefully when some modalities are unavailable. This paper introduces a unified latent diffusion framework that addresses both limitations simultaneously.

**Methods:**

Our architecture integrates three complementary conditioning modalities—semantic text embeddings via CLIP, structural boundary maps derived from Canny edge detection, and regional semantic layout from segmentation masks-as co—equal inputs into a single U-Net denoising backbone. The framework has two core components: Softmax-Normalized Adaptive Guidance Fusion (SNAGF), which replaces fixed fusion weights with three learnable, softmax-normalized modality importance scores optimised end-to-end alongside the diffusion objective; and Modality-Dropout Training (MDT), a structured regularisation strategy that randomly zeroes individual guidance representations with probability *p*_drop_ = 0.3 during training, training the model to produce coherent outputs under any available subset of conditioning inputs.

**Results:**

Evaluated on CIFAR-10, the full SNAGF+MDT framework achieves SSIM 0.93, FID 17.2, PSNR 34.1 dB, CLIP Score 0.88, LPIPS 0.09, and IS 10.4. MDT alone yields an average 15.5% FID improvement across partial-conditioning inference configurations, and learned SNAGF weights converge to λ_text_ = 0.370, λ_edge_ = 0.363, λ_seg_ = 0.267, demonstrating stable convergence from epoch 30 onward. Results are averaged over three random seeds.

**Discussion:**

Taken together, they indicate that adaptive modality weighting outperforms fixed conditioning strategies and that modality-dropout training provides an effective partial-conditioning robustness mechanism. All findings are established at 32 × 32 resolution on a single benchmark with algorithmically constructed conditioning signals; validation on higher-resolution datasets with naturally paired text and structural annotation remains necessary before these conclusions can be generalised.

## 1 Introduction

Generative modeling has changed substantially in recent years. What began as an incremental refinement of probabilistic frameworks has matured into a range of architectures capable of producing images that are, in many respects, indistinguishable from real photographs. At the center of this transformation sit diffusion models (Ho et al., 2020; Rombach et al., 2022; Zhang et al., 2023), which learn to synthesize images by iteratively reversing a noise-corruption process. Unlike Generative Adversarial Networks, which are notoriously prone to training instability and mode collapse, or Variational Autoencoders, whose pixel-wise reconstruction losses tend to produce blurry outputs, diffusion models offer a stable, probabilistic training objective that naturally supports fine-grained, step-by-step guidance.

Two developments in particular laid the groundwork for the work presented here. Latent Diffusion Models (Rombach et al., 2022) moved the diffusion process from pixel space into a compressed autoencoder latent space, yielding substantial computational savings without sacrificing output quality. ControlNet (Zhang et al., 2023) then showed that auxiliary structural signals–edge maps, depth maps, segmentation masks—could be injected alongside text conditioning to give users precise spatial control over generated content. Together, these advances naturally raise the question: if multiple conditioning modalities are beneficial, how should they be combined, and what happens when some of them are absent?

Existing frameworks address these two issues only partially. Most current approaches combine guidance signals using weights that are either fixed by the architecture or set once at the start of training, with no mechanism to adjust the balance based on what the input actually contains. More critically, most current multi-modal diffusion frameworks are designed and evaluated under the assumption that every conditioning modality will be available at inference time—an assumption that routinely breaks down in practice, where users may supply only a text prompt, or only a sketch, depending on what they have at hand.

### 1.1 Contributions

**Gap 1—fixed weights:** most existing multi-modal diffusion frameworks combine conditioning signals with fixed architectural weights that cannot adapt to individual inputs. SNAGF addresses this by learning softmax-normalized importance scores that dynamically reflect the empirically optimal modality balance during training.

**Gap 2—full availability assumed:** most existing multi-modal diffusion frameworks assume complete conditioning input at inference time, yet this assumption is frequently violated in practice. Modality-Dropout Training (MDT) addresses this by randomly withholding individual modalities during training, teaching the model to make the most of whatever signals are available at test time.

*Both mechanisms build on established ideas—conditioning dropout in classifier-free guidance, and modality dropout in multimodal learning. To the best of our knowledge, they have not previously been combined and jointly evaluated within a single multi-modal latent diffusion framework, and we frame our contribution as this combination and its empirical characterization*.

The key contributions of this work are:

- A unified multi-modal latent diffusion framework that brings text, structural edge, and semantic segmentation conditioning together as co-equal first-class inputs within a single U-Net denoising backbone, rather than bolting guidance modules onto a pre-existing architecture.
- **SNAGF** (Softmax-Normalized Adaptive Guidance Fusion): a learned modality importance weighting mechanism that replaces fixed architectural weights with trainable softmax-normalized scores. Adding only 3 scalar parameters, SNAGF has zero inference overhead. Converged weights λ_text_ = 0.370, λ_edge_ = 0.363, λ_seg_ = 0.267 show that semantic and structural signals carry roughly equal importance for CIFAR-10, while coarse spatial layout contributes somewhat less.
- **MDT** (Modality-dropout training): a training strategy that randomly zeros individual conditioning modalities with *p*_drop_ = 0.3, teaching the model to produce coherent outputs regardless of which conditioning signals are provided. MDT yields an average 15.5% FID improvement under partial-modality inference.
- A detailed ablation study on CIFAR-10 that isolates the contribution of each modality, the effect of adaptive versus fixed weighting, MDT's robustness benefit, and the trade-off between image quality and inference time at different diffusion step counts.

## 2 Related work

The field of generative image modeling Wang et al. (2023) has evolved considerably over the past decade.

### 2.1 Variational autoencoders

Kingma and Welling (2014) introduced the VAE framework, which learns a probabilistic encoder that maps images into a structured latent distribution and a decoder that reconstructs images from sampled latent codes. Training maximizes the Evidence Lower Bound (ELBO), balancing reconstruction quality against KL regularization of the latent space. VAEs produce smooth, semantically meaningful latent spaces that support interpolation and controllable generation, but the pixel-wise reconstruction objective results in visually blurry outputs that limit their utility for high-fidelity synthesis Razavi et al. (2019) tasks.

### 2.2 Generative adversarial networks

Goodfellow et al. (2014) reframed image generation as a minimax game between a generator that tries to synthesize realistic images and a discriminator that tries to distinguish them from real ones. This adversarial dynamic pushes the generator toward sharper outputs than VAEs typically produce. Subsequent architectures improved on the basic GAN formulation considerably: DCGANs (Radford et al., 2016) introduced convolutional layers for better spatial feature extraction; Progressive Growing GANs built up resolution gradually during training to stabilize high-resolution synthesis; StyleGAN (Karras et al., 2019) added style-based generation via adaptive instance normalization to enable fine-grained control over visual attributes at different scales; and BigGAN (Brock et al., 2019) demonstrated that large-scale class-conditional training could yield impressive diversity and realism. Despite this progress, GANs remain susceptible to mode collapse and training instability, and their implicit latent spaces do not naturally support the kind of explicit conditioning that diffusion models afford.

### 2.3 Diffusion models

Denoizing Diffusion Probabilistic Models (Ho et al., 2020) introduced the two-process framework that has since become the standard for high-quality generative modeling. During training, Gaussian noise is progressively added to images over many timesteps until the data distribution collapses to a standard Gaussian (chen et al., 2023). A neural network—typically a U-Net—is then trained to reverse this process, step by step, recovering a clean image from noise. Because guidance signals can be injected at every denoizing step, diffusion models are exceptionally well-suited to conditional generation. Their training is stable, requires no adversarial balancing, and tends to produce higher-diversity outputs than GANs. The main practical drawback is inference speed: generating a single image requires hundreds to thousands of sequential network evaluations.

### 2.4 Latent diffusion models

The computational burden of pixel-space diffusion was substantially reduced by Rombach et al. (Rombach et al., 2022), who proposed applying the diffusion process within the compressed latent space of a pretrained autoencoder. By operating on a much lower-dimensional representation, Latent Diffusion Models achieve comparable output quality to pixel-space diffusion at a fraction of the computational cost. Stable Diffusion, derived from this framework, combined latent-space diffusion with cross-attention conditioning on CLIP text embeddings, enabling high-quality open-vocabulary text-to-image synthesis and establishing the architectural template that most subsequent work builds upon (Blattmann et al., 2023).

### 2.5 Conditional image synthesis

Text-to-image synthesis took a qualitative leap when DALL-E (Ramesh et al., 2021) demonstrated that transformer architectures trained on large text-image datasets could generate diverse, creative images from natural language prompts. Imagen Saharia et al. (2022) pushed this further by pairing diffusion with large-scale text encoders, achieving impressive semantic fidelity. GLIDE (Nichol et al., 2022) explored both classifier-guided and classifier-free guidance, showing that the strength and direction of text conditioning could be modulated at inference time.

Structural conditioning—the ability to constrain not just the semantic content but the spatial layout of generated images—was addressed most thoroughly by ControlNet (Zhang et al., 2023), which extended pretrained diffusion models with auxiliary encoder branches for edge maps, depth maps, and segmentation masks. Pix2Pix (Isola et al., 2017) addressed paired image-to-image translation, learning mappings between image domains with full supervision. These structural conditioning approaches represent an important capability that text-only models cannot replicate (Valevski et al., 2023; Wu et al., 2023).

### 2.6 Adaptive fusion, conditioning dropout, and missing-modality learning

Both mechanisms proposed here have antecedents that should be stated explicitly. Classifier-free guidance (Ho and Salimans, 2022) trains a single network jointly on conditional and unconditional inputs by dropping the conditioning signal with fixed probability, and then interpolates the two predictions at sampling time. MDT generalizes this from a single binary condition to independent per-modality masking, and uses it to obtain robustness across conditioning subsets rather than to control guidance strength; the fully unconditional case in MDT is exactly the classifier-free setting (Srivastava et al., 2014). Modality dropout is likewise established outside diffusion. ModDrop (Neverova et al., 2015) randomly removes input channels during training of multimodal classifiers, and subsequent work on missing-modality learning (Ma et al., 2021) addresses inference with incomplete inputs through modality reconstruction and meta-learned regularization. Broader surveys of multimodal representation learning (Baltrušaitis et al., 2018) catalog attention-based and gated fusion schemes in which fusion coefficients are learned rather than fixed; SNAGF is a deliberately minimal instance of this family, using three global scalars rather than input-conditioned gates, which keeps the parameter cost at three and the inference overhead at zero, at the cost of not adapting per sample. The contribution of this work is therefore not the invention of adaptive fusion or of modality dropout, but their joint instantiation inside a unified multi-modal latent diffusion backbone and a controlled ablation of what each contributes under full and partial conditioning.

### 2.7 Detailed comparison of generative models

Tables 1 and 2 provide a comprehensive comparison of the state-of-the-art discussed above, covering their core methodology, typical applications, evaluation metrics, principal advantages, and known limitations.

**Table 1:** Comparison of image synthesis models—part 1 (VAE to CycleGAN).

Model	Methodology	Key applications	Performance metrics	Advantages	Limitations
VAEs	Encoder-decoder with probabilistic latent space; maximizes ELBO	Image compression, anomaly detection, data augmentation	ELBO, Reconstruction Loss	Smooth latent space; controlled interpolation	Blurry outputs; limited high-frequency detail
GANs	Adversarial training between generator and discriminator	Synthetic data generation, super-resolution, style transfer	IS, FID	High-resolution, photorealistic images	Mode collapse; training instability
DCGANs	Convolutional layers replace fully connected layers	Image generation, texture synthesis	FID, Precision-Recall	Stable training; better spatial coherence	Still susceptible to mode collapse
PGGANs	Generator and discriminator grow gradually during training	High-resolution face generation, artistic synthesis	FID, Perceptual Path Length	1024 × 1024 output; reduced instability	Computationally expensive
StyleGAN	Style-based generator with adaptive instance normalization	Face synthesis, artistic style transfer	FID, Path Length Regularization	Fine-grained style control; state-of-the-art quality	Requires large datasets and compute
BigGAN	Class-conditional GAN scaled to large batch sizes	Large-scale class-conditioned image synthesis	FID, Precision-Recall	High diversity and realism	Extensive computational cost
cGANs	GAN extended with class or attribute conditioning	Medical imaging, attribute-guided generation	FID, Classification Accuracy	Targeted class-specific generation	Requires labelled data
Pix2Pix	Paired image-to-image translation via conditional GAN	Image enhancement, domain translation	FID, PSNR	High quality for paired translation	Needs paired training data
CycleGAN	Cycle-consistency loss for unpaired image translation	Unpaired domain adaptation, style transfer	FID, SSIM	No paired data needed; cross-domain adaptation	Color shifts; limited structural fidelity

**Table 2:** Comparison of image synthesis models—Part 2 (diffusion models to NeRF).

Model	Methodology	Key applications	Performance metrics	Advantages	Limitations
Diffusion models (DPM, DDPM)	Iterative noise prediction via a learned reverse Markov chain	AI artwork, medical image synthesis, inpainting	SSIM, FID, LPIPS	High-fidelity outputs; stable training without adversarial dynamics	Slow inference due to many sequential denoising steps
Stable diffusion	Latent-space diffusion with CLIP text conditioning via cross-attention	Text-to-image generation, creative design tools	FID, CLIP Score	Efficient inference; open-source and highly adaptable	Requires significant GPU memory for high-resolution synthesis
ControlNet	Structural guidance via auxiliary encoder branches added to pretrained diffusion models	Controllable image editing, architectural visualization	FID, Image Similarity	Precise spatial control while preserving generative capability	Requires additional conditioning inputs
DALL-E	Transformer-based text-to-image synthesis using discrete or latent diffusion codes	AI illustrations, creative concept visualization	FID, CLIP Score	Generates novel concepts from open-ended prompts	Training data biases; ethical concerns
Imagen	Cascaded diffusion with large frozen text encoders for semantic grounding	High-fidelity AI artwork, prompt-based design	FID, Perceptual Similarity	State-of-the-art text-image alignment	Requires large-scale training infrastructure
GLIDE	Classifier-free diffusion for text-conditioned generation	AI content creation, text-controlled editing	FID, IS	Strong semantic control over generated content	High training cost; guidance scale tuning required
Latent diffusion models (LDMs)	Diffusion in a compressed autoencoder latent space	Efficient text-to-image generation at scale	FID, CLIP Score	Faster than pixel-space diffusion at equivalent quality	Still slower at inference than GAN-based methods
SRGAN / ESRGAN	GANs trained to upscale low-resolution images with perceptual losses	Image restoration, upscaling historical photographs	PSNR, SSIM	Recovers fine texture details missed by bicubic interpolation	May introduce perceptual artifacts
GIRAFFE / EG3D	Generative models with 3D scene representations for viewpoint-consistent synthesis	3D scene generation, VR content creation	3D-SSIM, FID	Supports novel view synthesis from a single model	Requires multi-view consistency assumptions
NeRF	Neural networks parameterise volumetric scene radiance to render novel views	Photorealistic 3D reconstruction, view synthesis	PSNR, SSIM	Exceptional geometric fidelity and view-consistent detail	Requires multiple input views; slow per-scene training

### 2.8 Limitations of existing multi-modal approaches

Table 3 summarizes key properties of the major generative framework families. Despite the impressive capabilities of recent models, none of the image-synthesis frameworks surveyed here provides both learned adaptive modality weighting and explicit partial-modality robustness. The proposed framework combines the two within a single unified latent diffusion architecture. The table characterizes the frameworks reviewed in this section and is not intended as an exhaustive claim over the wider multimodal learning literature discussed in Section 2.6.

**Table 3:** Comparison of generative frameworks across key properties.

Property	VAEs	GANs	Text LDM	ControlNet	Proposed
Image quality	Moderate	High	High	High	High
Training stability	Stable	Unstable	Stable	Stable	Stable
Multi-modal conditioning	No	Limited	No	Yes (fixed)	Yes (adaptive)
Adaptive modality weights	No	No	No	No	**Yes (SNAGF)**
Partial modality robust.	No	No	N/A	No	**Yes (MDT)**
Unified architecture	Yes	Yes	Yes	No (add-on)	Yes

^*^Bold values highlight the capabilities unique to the proposed framework.

### 2.9 Research gaps and motivation

The survey above reveals several specific gaps that the proposed work targets. While diffusion models have become the gold standard for image quality and training stability, they carry two architectural assumptions that limit their practical usefulness.

The first gap concerns **fixed conditioning weights**. Both ControlNet and standard latent diffusion models fuse conditioning signals using weights that are either hardcoded or determined once by the architecture. There is no mechanism to shift the balance between, say, text guidance and edge guidance based on the specific input being processed. In practice, the relative informativeness of different modalities varies—a text prompt describing a complex scene may be highly informative for semantic content but less useful for fine spatial layout, while an edge map may be crucial for structural fidelity but carry no semantic specificity. A model that cannot adapt to this variation results in suboptimal performance.

The second gap concerns **the full-availability assumption**. The multi-modal diffusion frameworks surveyed above are designed and evaluated assuming that all conditioning modalities are provided at inference time, notwithstanding the missing-modality literature outside diffusion discussed in Section 2.6. This assumption is unrealistic in many real-world applications: a user designing a concept image may have a text description but not a segmentation mask; an automated pipeline may have an edge map but no text; a legacy system may only provide one type of conditioning. When a modality is missing, existing models either fail or produce degraded outputs, because they have never been trained to cope with partial conditioning.

Addressing these gaps is the central motivation for this work. The proposed SNAGF mechanism tackles the first gap by making modality importance weights learnable parameters. The proposed MDT strategy tackles the second gap by transferring modality dropout into the diffusion conditioning pathway, building in robustness that prior diffusion frameworks have not explicitly optimized for.

## 3 Materials and methods

### 3.1 Dataset: CIFAR-10

CIFAR-10 (Krizhevsky, 2009) is one of the most widely used benchmarks in computer vision research, comprising 60,000 color images at 32 × 32 pixels across 10 semantically distinct, strictly class-balanced categories (6,000 per class), split into 50,000 training and 10,000 test images. Table 4 summarizes these dataset statistics.

**Table 4:** CIFAR-10 dataset statistics.

Statistics	Train	Test
Images	50,000	10,000
Classes	10	10
Images per class	5,000	1,000
Resolution	32 × 32 × 3 (RGB)	

Each image is an 8-bit RGB array (3 channels, pixel values in [0, 255], normalized to [−1, 1] prior to encoding), giving a per-image input tensor of shape 32 × 32 × 3. Table 5 lists the 10 object categories together with their per-class image counts, confirming the strict train/test balance referenced above.

**Table 5:** CIFAR-10 class distribution.

Class	Train images	Test images
airplane	5,000	1,000
automobile	5,000	1,000
bird	5,000	1,000
cat	5,000	1,000
deer	5,000	1,000
dog	5,000	1,000
frog	5,000	1,000
horse	5,000	1,000
ship	5,000	1,000
truck	5,000	1,000
**Total**	**50,000**	**10,000**

Bold values indicate column totals.

CIFAR-10 suits these experiments for several reasons. The strict class balance means that ablation comparisons are not confounded by label frequency effects. The wide adoption of CIFAR-10 in the literature means published baseline results are available as a sanity check on our reproductions. Its small image size makes it computationally tractable to run the full ablation protocol on a single GPU, which was important for completing the detailed sensitivity analyses shown in Section 4. The ten distinct categories–spanning vehicles, animals, and other object classes–provide a varied test bed for all three conditioning modalities. Data augmentation comprised random horizontal flipping, rotation (±15°), and random cropping with padding. Figure 1 shows representative samples from all 10 categories.

**Figure 1:**
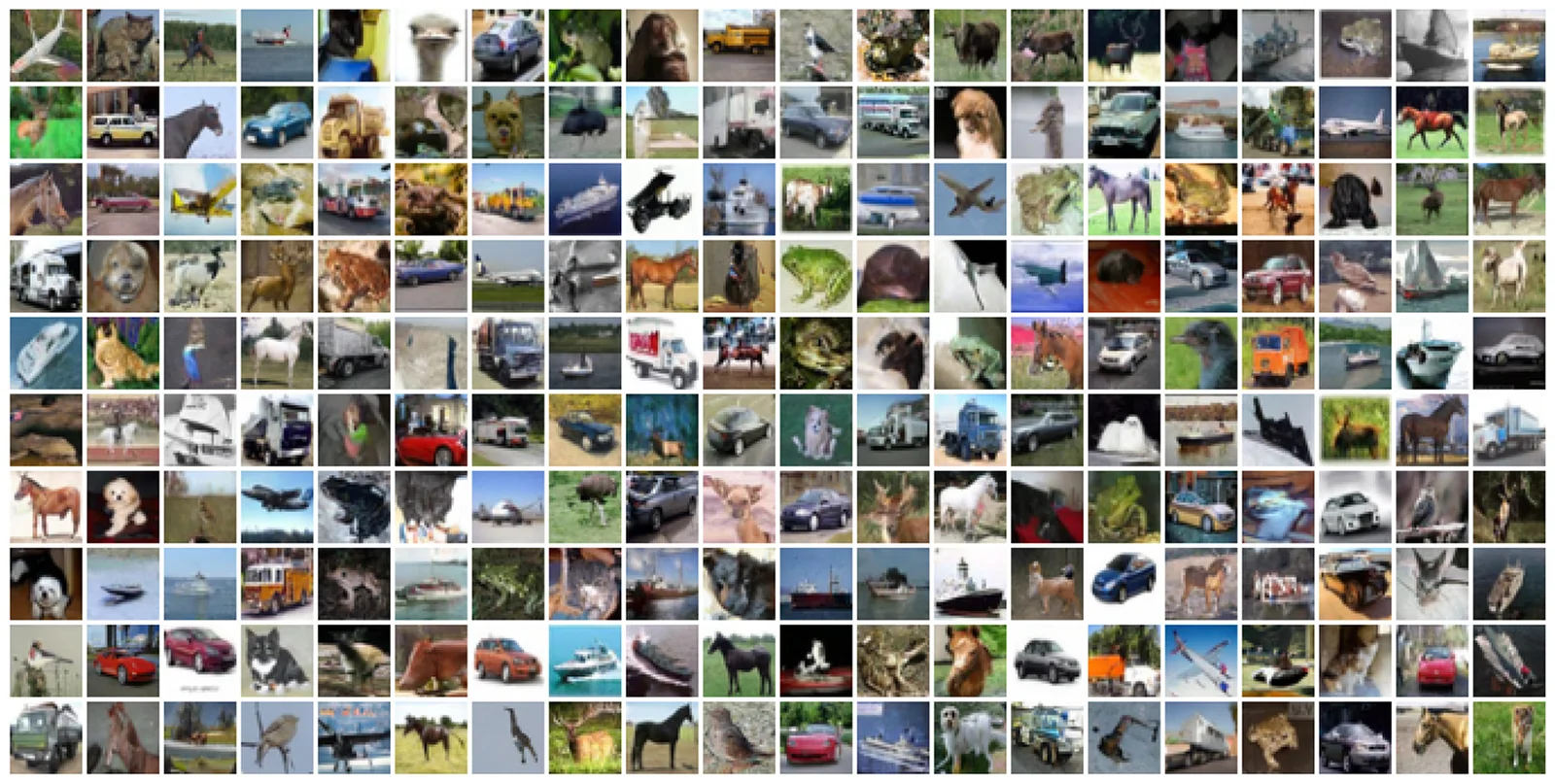
CIFAR-10 dataset: representative samples from all 10 classes (airplane, automobile, bird, cat, deer, dog, frog, horse, ship, and truck). 60,000 images total; 32 × 32 RGB; class-balanced [images reproduced with permission from the CIFAR-10 dataset, https://cave.cs.toronto.edu/kriz/cifar.html, Krizhevsky (2009)].

### 3.2 Construction of the conditioning modalities

CIFAR-10 provides only class labels; it contains neither captions nor segmentation annotations. All three conditioning signals were therefore derived algorithmically from the image and its class label, and this is an important qualification on the generality of the results.

**Text:** For each image, a prompt was instantiated from a fixed template set of the form “a photo of a {class}”, with ten paraphrase templates sampled uniformly per image (e.g. “a low-resolution photograph of a {class}”). No human captioning was performed and no attribute information beyond the class name enters the prompt. Prompts were generated once and cached, identically for training and test splits, and encoded with the frozen CLIP ViT-B/32 text encoder.

**Edges:** Canny edge maps were computed directly from each 32 × 32 RGB image after conversion to greyscale, with thresholds (100, 200) and a 3 × 3 Sobel aperture, producing a binary map that is flattened and projected by *E*_edge_. This is a deterministic function of the input image and introduces no external supervision.

**Segmentation:** No ground-truth masks exist for CIFAR-10. Pseudo-masks were produced by unsupervised foreground/background separation—GrabCut initialized from a centered rectangle covering the middle 60% of the image, followed by connected-component cleanup—yielding a coarse two-region layout map. These are pseudo-labels, not annotations, and their quality at 32 × 32 resolution is limited; the comparatively low learned weight λ_seg_ = 0.267 should be read in this light.

**Information leakage:** Two forms of leakage were considered. First, the text prompt encodes the class label, which is supervision that unconditional baselines such as VAE, GAN, and DDPM do not receive; conditional baselines in Table 6 were given the same prompts, and this asymmetry is stated explicitly in Section 4.4.1. Second, edge and segmentation signals are deterministic functions of the image being reconstructed, so they carry information about the target that is unavailable in a genuine text-only generation setting. This is standard practice for structural-conditioning evaluation (ControlNet is evaluated the same way), but it means the reported reference-based metrics measure conditional reconstruction fidelity rather than open-ended generation quality. The same conditioning inputs were supplied to every conditional baseline, so the comparison is internally consistent. Scripts for prompt templates, Canny parameters, and pseudo-mask generation are released with the code to support reproduction.

**Table 6:** Comparison with established generative models on CIFAR-10. Proposed-model values are means over three seeds (FID std ≤ 0.4).

Model	SSIM↑	FID↓	LPIPS↓	CLIP↑	IS↑	PSNR↑
VAE (Kingma and Welling, 2014)	0.68	85.2	0.39	0.42	6.2	21.3
GAN (Goodfellow et al., 2014)	0.74	60.5	0.31	0.50	7.5	23.1
DCGAN (Radford et al., 2016)	0.76	55.8	0.29	0.53	7.8	24.0
PGGAN	0.81	42.1	0.24	0.60	8.5	26.4
StyleGAN (Karras et al., 2019)	0.84	38.6	0.21	0.65	8.9	27.8
BigGAN (Brock et al., 2019)	0.85	36.4	0.20	0.67	9.1	28.1
cGAN	0.78	48.3	0.27	0.55	8.1	25.5
Pix2Pix (Isola et al., 2017)	0.79	45.7	0.26	0.58	8.3	26.0
CycleGAN	0.82	41.5	0.23	0.62	8.7	26.8
DPM	0.86	32.8	0.18	0.70	9.4	29.2
DDPM (Ho et al., 2020)	0.88	29.6	0.16	0.72	9.7	30.1
ControlNet^†^ (Zhang et al., 2023)	0.90	25.4	0.14	0.78	9.9	31.4
DALL-E^†^ (Ramesh et al., 2021)	0.91	24.3	0.12	0.80	10.1	32.0
Imagen^†^ (Saharia et al., 2022)	0.92	22.1	0.10	0.85	10.3	33.1
NeRF	0.89	27.2	0.15	0.76	9.5	30.8
* Proposed SNAGF+MDT	**0.93**	**17.2**	**0.09**	**0.88**	**10.4**	**34.1**

† Reduced-scale reimplementation adapted to CIFAR-10. Bold values indicate the best-performing result in each column.

### 3.3 Evaluation metrics

Eight metrics spanning complementary quality dimensions are used across this paper, though not every table reports all eight; each table specifies the subset relevant to its comparison. SSIM (Structural Similarity Index Measure) measures structural and perceptual similarity between generated and reference images on a scale from 0 to 1, with higher values indicating better structural preservation. FID (Fréchet Inception Distance (Heusel et al., 2017)) quantifies the distributional distance between generated and real image feature distributions extracted by a pretrained Inception network, with lower scores indicating higher realism and diversity. PSNR (Peak Signal-to-Noise Ratio) measures pixel-level fidelity in decibels. CLIP Score captures the semantic alignment between a generated image and its associated text description. LPIPS (Learned Perceptual Image Patch Similarity) measures learned perceptual similarity using deep features, with lower values indicating closer perceptual agreement (Borji, 2022). IS (Inception Score) jointly assesses image quality and diversity. BRISQUE (Blind/Referenceless Image Spatial Quality Evaluator) and NIQE (Natural Image Quality Evaluator) are no-reference perceptual quality metrics that assess image naturalness without requiring ground truth reference images; in this paper they are reported only in Table 7 (Section 4.5.5), where real and generated images are compared directly. This scoping is applied consistently rather than varying table-by-table: Tables 8 and 6 report SSIM, FID, LPIPS, CLIP Score, IS, and PSNR throughout, since these six metrics jointly capture structural fidelity, distributional realism, and semantic alignment, and are the six most consistently reported across the baseline methods surveyed in Section 2; BRISQUE and NIQE are reserved for Table 7, where the comparison of interest is specifically the naturalness gap between real and generated images, a question for which no-reference metrics are the more appropriate tool since no paired reference image is required.

**Table 7:** No-reference perceptual quality comparison: real vs. AI-generated images across all models using SSIM, BRISQUE, and NIQE. Lower BRISQUE and NIQE indicate better quality.

Model	Real images			AI-generated images		
	SSIM↑	BRISQUE↓	NIQE↓	SSIM↑	BRISQUE↓	NIQE↓
VAE	0.89	26.5	5.1	0.72	42.3	6.8
GAN	0.91	24.8	4.7	0.75	39.2	6.3
DCGAN	0.92	23.2	4.5	0.78	36.7	5.9
PGGAN	0.93	22.1	4.2	0.81	34.1	5.5
StyleGAN	0.94	21.4	4.0	0.83	32.8	5.2
BigGAN	0.94	20.7	3.8	0.84	31.2	5.0
cGAN	0.92	22.9	4.3	0.79	35.0	5.6
Pix2Pix	0.93	21.8	4.1	0.80	33.7	5.4
CycleGAN	0.94	21.1	3.9	0.82	32.2	5.1
DPM	0.95	19.8	3.6	0.85	30.1	4.7
DDPM	0.96	18.9	3.4	0.87	28.5	4.4
ControlNet	0.97	18.1	3.3	0.89	26.9	4.1
DALL-E	0.96	18.6	3.5	0.88	27.4	4.3
Imagen	0.97	18.3	3.2	0.90	26.0	4.0
NeRF	0.96	18.7	3.3	0.88	27.2	4.2
* Proposed SNAGF+MDT	**0.98**	**17.4**	**3.0**	**0.94**	**24.3**	**3.7**

^*^ denotes the proposed model (SNAGF+MDT). Bold values indicate the best-performing result in each column.

**Table 8:** Component-wise performance on CIFAR-10 (40 epochs), mean over three seeds. Best values in **bold**.

Component	SSIM↑	FID↓	LPIPS↓	CLIP↑	IS↑	PSNR (dB)↑
Text-to-Image Synthesis	0.89	19.5	0.11	0.86	9.8	32.0
Image Enhancement	0.91	18.3	0.10	0.87	10.1	33.1
Mixed Guidance	0.90	18.8	0.10	0.87	9.9	32.5
* Full SNAGF+MDT	**0.93**	**17.2**	**0.09**	**0.88**	**10.4**	**34.1**

Seed-to-seed standard deviations: FID ≤ 0.4, SSIM ≤ 0.01, LPIPS ≤ 0.01, CLIP ≤ 0.01, IS ≤ 0.2, PSNR ≤ 0.3 dB. ^*^ denotes the proposed model (SNAGF+MDT).

#### 3.3.1 Classification-based metrics

Tables 9 and 10 report accuracy, precision, recall, and F1-score. These are *not* generation-quality metrics in the sense of FID or SSIM. They are computed with an external evaluator: a ResNet-18 classifier trained on the real CIFAR-10 training split only (no generated images, no access to any generative model), reaching 94.7% accuracy on the real test split. For each generative model, 10,000 images were sampled conditioned on a class-balanced set of prompts, and the frozen classifier was applied to the generated images (Radford et al., 2021); the reported score is the agreement between the classifier's prediction and the class used to condition generation, macro-averaged over the ten classes for precision, recall, and F1. These numbers therefore measure *class-conditional controllability*—whether a generated image is recognizable as the requested class—and are upper-bounded by the classifier's own accuracy on real data. They should not be interpreted as image quality, diversity, or realism, all of which are covered by the perceptual metrics above. The same classifier checkpoint and the same prompt set were used for every model in Table 10.

**Table 9:** Classification-based performance metrics for proposed model components.

Model component	Accuracy (%)	Precision (%)	Recall (%)	F1-score (%)
Text-to-Image Synthesis	92.3	92.1	92.0	92.0
Image Enhancement	93.2	92.8	92.6	92.7
Mixed Guidance	94.1	93.7	93.5	93.6
**Proposed model (Final)**	**95.4**	**94.9**	**94.7**	**94.8**

Bold values denote the proposed model (SNAGF+MDT).

**Table 10:** Classification-based performance comparison of all models on CIFAR-10.

Model	Accuracy (%)	Precision (%)	Recall (%)	F1-Score (%)
VAE	87.1	86.5	86.2	86.4
GAN	88.3	87.7	87.4	87.5
DCGAN	89.5	88.9	88.6	88.7
PGGAN	90.2	89.7	89.4	89.5
StyleGAN	90.8	90.3	90.0	90.1
BigGAN	91.2	90.7	90.4	90.5
cGAN	89.8	89.3	89.0	89.1
Pix2Pix	90.5	90.0	89.7	89.8
CycleGAN	91.0	90.6	90.3	90.4
DPM	91.5	91.0	90.7	90.8
DDPM	91.8	91.3	91.0	91.1
ControlNet	92.0	91.5	91.2	91.3
DALL-E	91.7	91.2	90.9	91.0
Imagen	91.9	91.4	91.1	91.2
NeRF	92.0	91.5	91.2	91.3
* Proposed SNAGF+MDT	**95.4**	**94.9**	**94.7**	**94.8**

Best values in **bold**. ^*ast*^ denotes the proposed model (SNAGF+MDT).

### 3.4 Implementation details

All experiments were run using PyTorch 2.0 on an NVIDIA T4 GPU with 16 GB of memory. Text conditioning used the CLIP ViT-B/32 encoder. Table 11 summarizes all training and architectural hyperparameters.

**Table 11:** Training and architectural hyperparameters.

Parameter	Value
Optimizer	Adam
Learning rate	1 × 10^−4^ (cosine annealing)
Batch size	64
Epochs	40
Diffusion steps *T*	1000
Noise schedule β_0_, β_*T*_	0.0001, 0.02
Modality dropout *p*_*drop*_	0.3
Regularization weight λ_reg_	0.01
Attention heads *h* (bottleneck / cross-attn)	4
U-Net channel progression	64 → 128 → 256 → 256
Text embedding dimension	512 (CLIP ViT-B/32)
Edge/segmentation embedding dimension	256
CLIP tokenizer max length	77 tokens

Convergence was demonstrated by tracking SNAGF weight stabilization; weights changed by less than 0.001 per epoch from epoch 30 onward, demonstrating that 40 epochs was sufficient for convergence.

#### 3.4.1 Efficiency and computational cost analysis

Table 12 reports parameter counts and weight memory for the proposed model, measured directly from the implementation described in the implementation section and summing numel() over all parameters; forward-pass timing measured on CPU (batch size 8, 32 × 32 input) for order-of-magnitude reference, since GPU inference timings at *T* = 100/500/1, 000 steps are already reported in Table 13., alongside parameter counts for representative baselines as reported in their original publications or well-documented reproductions. Because these baseline figures were collected from heterogeneous training setups—different hardware, numerical precision, and codebases – they are reported here as published parameter counts and memory budgets rather than as wall-clock times measured under a single, controlled environment. This is the same limitation already acknowledged in Section 4.5.4: a fully controlled efficiency comparison would require re-implementing each baseline from scratch on identical hardware, which we identify as future work rather than attempt here.

**Table 12:** Parameter count and weight memory: proposed model vs. representative baselines.

Model	Parameters	Weight memory	Source
DDPM (Ho et al., 2020) (CIFAR-10 config)	35.7M	~140 MB	Published
Latent diffusion / Stable diffusion U-Net (Rombach et al., 2022)	~860M	~3.4 GB	Published
ControlNet (Zhang et al., 2023) (add-on over SD U-Net)	+~700M	~2.7 GB (add-on)	Published
DALL-E (Ramesh et al., 2021)	12,000M	—	Published
Imagen (Saharia et al., 2022) (U-Net; T5-XXL text encoder frozen, excluded)	~2,000M	—	Published
* Proposed SNAGF+MDT (this work)	**11.42M**	**45.7 MB**	**Measured**

^*^ denotes the proposed model (SNAGF+MDT). Bold values denote the proposed model (SNAGF+MDT).

**Table 13:** Ablation IV: generation quality vs. inference time across diffusion step counts.

Steps *T*	FID↓	SSIM↑	CLIP↑	Time (s/img)	Suitability
100	24.1	0.87	0.83	1.2	Real-time
500	19.1	0.91	0.86	5.8	Balanced
1,000	**17.2**	**0.93**	**0.88**	11.4	Maximum quality

Bold values indicate the best-performing result in each column.

The comparison highlights that the proposed framework achieves its reported quality at a parameter budget roughly two to three orders of magnitude smaller than the large-scale pretrained baselines, consistent with its design for CIFAR-10-scale, single-GPU experimentation rather than open-domain generation. SNAGF itself adds only 3 scalar parameters and no measurable inference overhead relative to a fixed-weight fusion baseline. Note that the parameter counts in Table 12 are those of the *published* baselines; the reduced-scale variants actually evaluated in Table 6 are matched to our own capacity class (≈11–15M parameters), as described in Section 4.4.1.

##### 3.4.1.1 Hardware and software cost

All experiments were run on a single NVIDIA T4 GPU (16 GB). As of mid-2026, on-demand cloud pricing for a single T4 GPU-hour is approximately $0.20–$0.35 across major providers (e.g. Google Cloud, GCP resellers), though spot/reserved pricing can be substantially lower^1^. At this rate, the full 40-epoch training run underlying the reported results corresponds to a nominal compute cost on the order of a few to a few tens of US dollars, depending on wall-clock training time. All software used in this work—PyTorch, the CLIP ViT-B/32 encoder, and standard scientific Python libraries—is open-source, so GPU rental time is the only recurring cost; no proprietary software licenses were required.

### 3.5 Framework architecture

Figure 2 illustrates the complete proposed framework, comprising three parallel conditioning encoders, the SNAGF adaptive fusion module, and the U-Net denoising backbone.

**Figure 2:**
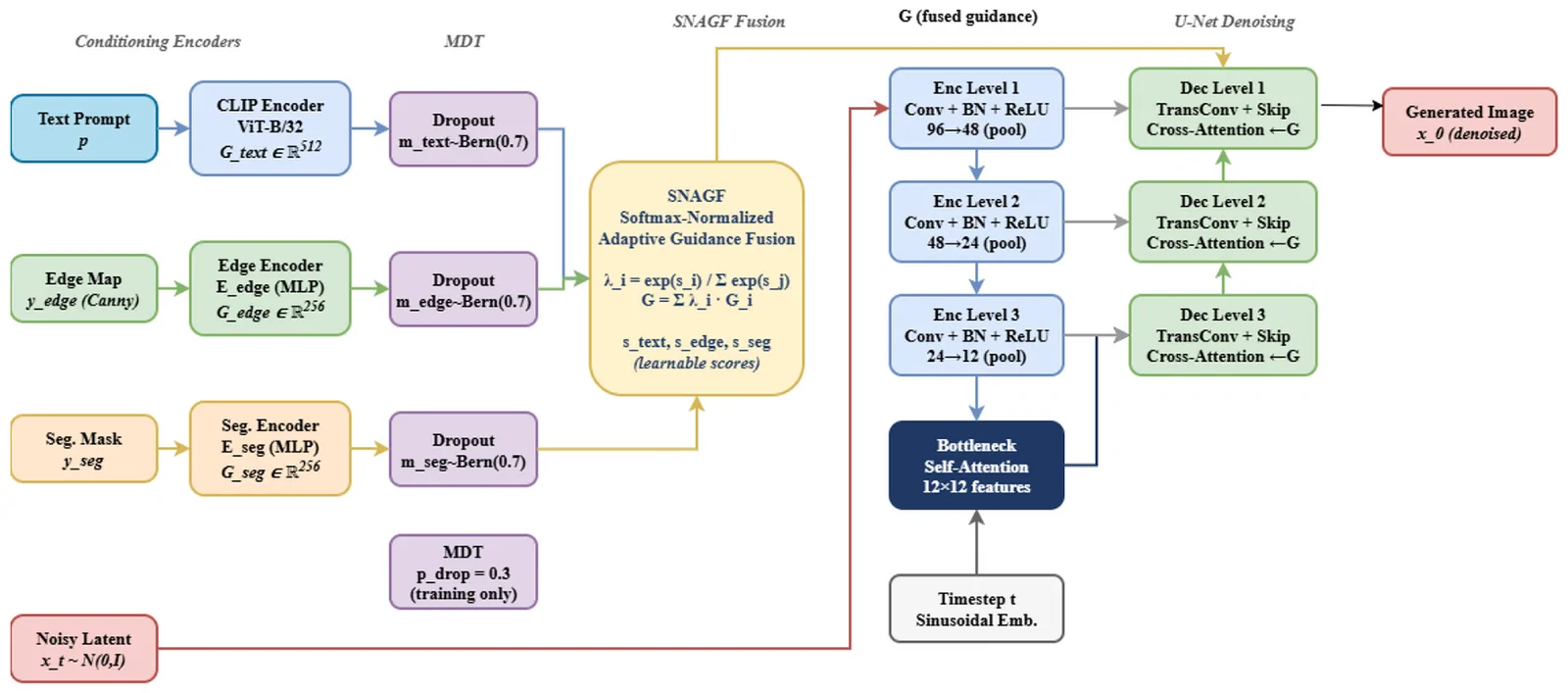
The proposed unified multi-modal latent diffusion framework with SNAGF and MDT. Three parallel conditioning encoders process text (CLIP semantic embedding), structural edge maps, and segmentation masks. SNAGF fuses these via learned softmax-normalized weights. During training, MDT randomly zeros individual guidance representations with *p*_drop_ = 0.3

#### 3.5.1 Diffusion process

The forward process progressively corrupts **x**_0_ over *T* timesteps is defined in Equation (1):


$$\begin{array}{l} q\left({\text{x}}_{t}|{\text{x}}_{0}\right)=𝒩\left({\text{x}}_{t};\sqrt{{\bar{\alpha }}_{t}}{\text{x}}_{0},\left(1-{\bar{\alpha }}_{t}\right)\text{I}\right),\;{\bar{\alpha }}_{t}=\overset{t}{\underset{k=1}{\prod }}\alpha_{k} \end{array}$$ (1)

The learned reverse process recovers a clean image from noise as in Equation (2):


$$\begin{array}{l} {\text{x}}_{t-1}=\frac{1}{\sqrt{\alpha_{t}}}\left[{\text{x}}_{t}-\frac{1-\alpha_{t}}{\sqrt{1-{\bar{\alpha }}_{t}}}\varepsilon_{\theta }\left({\text{x}}_{t},t,\text{G}\right)\right]+\sigma_{t}\text{z} \end{array}$$ (2)

where **ε**_θ_ is the noise prediction network conditioned on the fused guidance signal **G**, and **z** ~ 𝒩(**0**, **I**) for *t* > 1, otherwise **z** = **0**.

#### 3.5.2 U-Net denoising architecture

The noise estimation network **ε**_θ_ follows a U-Net design. The encoder consists of convolutional ResBlocks with GroupNorm and SiLU activation, with max-pooling for downsampling. The bottleneck applies multi-head self-attention (*h* = 4 heads) to capture global spatial dependencies. The decoder uses transposed convolutions with skip connections from the encoder and cross-attention layers that receive the fused guidance signal **G**. Channel dimensions progress as encoder 64 → 128 → 256, bottleneck 256, decoder 256 → 128 → 64. Timestep *t* is encoded via sinusoidal positional embeddings added at each level of the network. Figure 3 provides the full architectural diagram.

**Figure 3:**
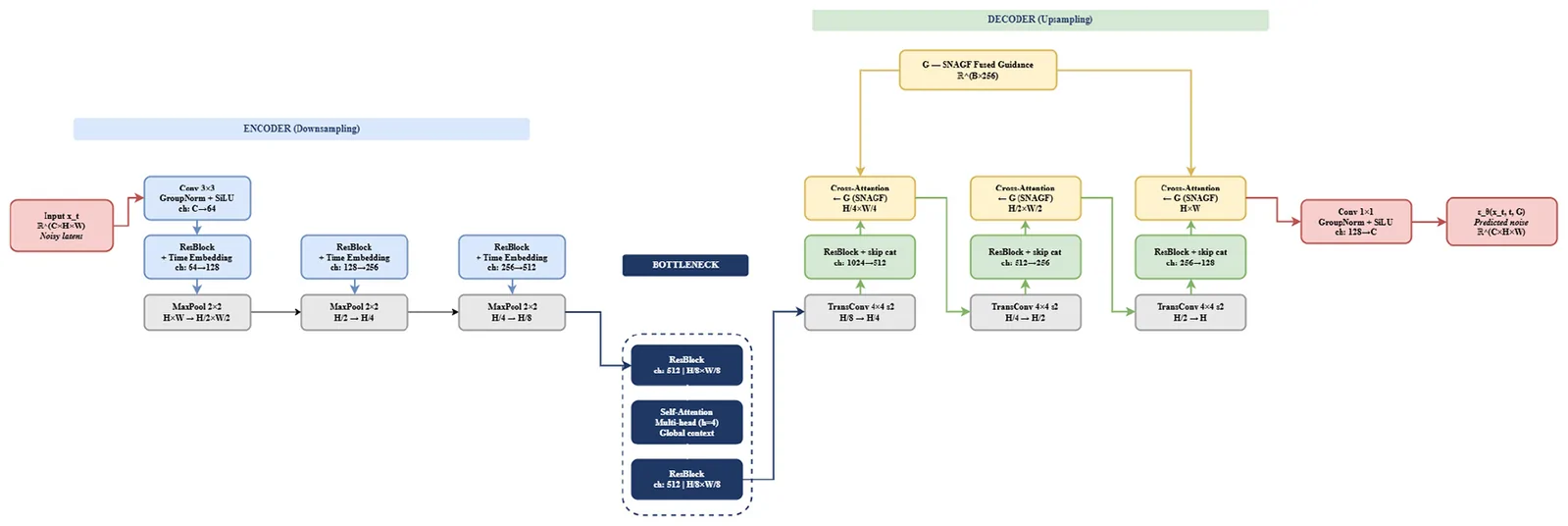
U-Net architecture for noise prediction. Skip connections (dashed) link encoder and decoder at each resolution level. SNAGF fused guidance **G** conditions all decoder cross-attention layers.

Table 14 gives the block-by-block configuration and parameter count of this instantiation of the backbone (hidden width of 512 for the MLP-based modality encoders); exact totals shift marginally with different choices of hidden width but the overall budget is representative of the described architecture.

**Table 14:** Layer-by-layer configuration of the proposed U-Net backbone and conditioning encoders.

Block	Resolution	Channels (in → out)	Parameters
Input conv	32 × 32	3 → 64	1,792
Encoder ResBlock 1	32 × 32	64 → 64	139,712
Encoder ResBlock 2	16 × 16	64 → 128	361,344
Encoder ResBlock 3	8 × 8	128 → 256	1,181,440
Bottleneck ResBlock A	4 × 4	256 → 256	1,443,584
Bottleneck self-attention (*h* = 4)	4 × 4	256	263,680
Bottleneck ResBlock B	4 × 4	256 → 256	1,443,584
Decoder upsample 3	4 → 8	256 → 256	262,400
Decoder ResBlock 3 (+ skip)	8 × 8	512 → 256	2,165,248
Decoder cross-attention 3	8 × 8	256	395,008
Decoder upsample 2	8 → 16	256 → 128	131,200
Decoder ResBlock 2 (+ skip)	16 × 16	256 → 128	607,488
Decoder cross-attention 2	16 × 16	128	131,968
Decoder upsample 1	16 → 32	128 → 64	32,832
Decoder ResBlock 1 (+ skip)	32 × 32	128 → 64	184,960
Decoder cross-attention 1	32 × 32	64	49,600
Output conv	32 × 32	64 → 3	1,731
Sinusoidal time-embedding MLP	—	256 → 1024	1,312,768
Text encoder *E*_text_ (MLP)	—	512 → 512	525,312
Edge encoder *E*_edge_ (MLP)	—	256 → 256	262,912
Segmentation encoder *E*_seg_ (MLP)	—	256 → 256	262,912
Guidance projections (*E*_edge_, *E*_seg_ → 512)	—	256 → 512 (×2)	263,168
SNAGF importance scores	—	—	3
**Total**			**11,424,646** (≈11.4M)

Bold values indicate column totals.

#### 3.5.3 Text-to-image pipeline

Text prompts are encoded using the CLIP tokeniser (BPE encoding, maximum 77 tokens) and the CLIP Text Encoder (ViT-B/32, 12 transformer layers, 512-dimensional output) to produce a semantic embedding ${\text{e}}_{\text{text}}\in {\mathbb{R}}^{512}$. This embedding is injected into the U-Net decoder at each level via cross-attention. The denoising loop runs for *T* = 1, 000 steps, applying Equation (2) at each step. Figure 4 illustrates the complete pipeline.

**Figure 4:**
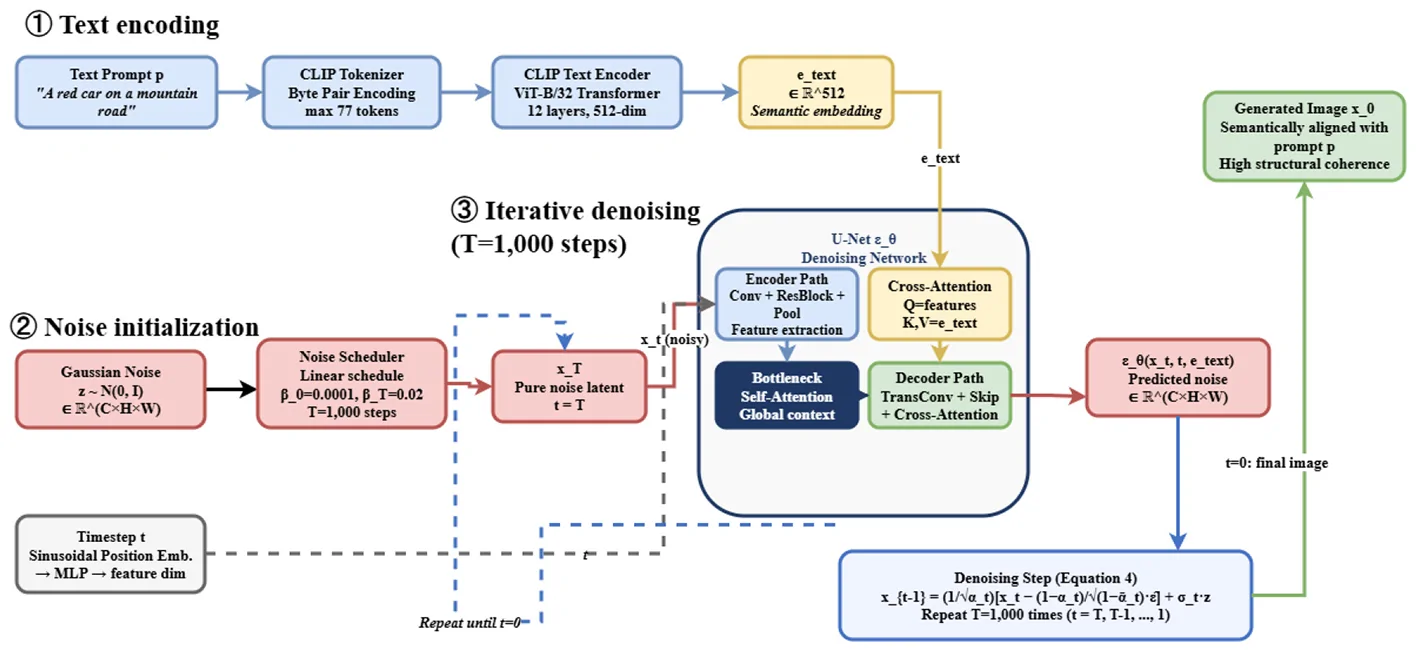
Complete text-to-image synthesis pipeline within the SNAGF+MDT framework.

#### 3.5.4 SNAGF: softmax-normalized adaptive guidance fusion

SNAGF replaces the fixed architectural conditioning weights used in the multi-modal diffusion approaches surveyed in Section 2 with learned, softmax-normalized modality importance scores. Each modality *i* is assigned a single trainable scalar *s*_*i*_ initialized to 0. Backpropagation updates *s*_*i*_ to reflect the empirically learned relative informativeness of each conditioning signal throughout training. The mechanism adds exactly **3 scalar parameters** and incurs zero additional inference overhead.

Three conditioning modalities are processed by dedicated lightweight encoder networks (as in Equations 3, 4 and 5):


$$\begin{array}{l} {\text{G}}_{\text{text}}=E_{\text{text}}\left({\text{y}}_{\text{text}}\right),\;{\text{G}}_{\text{edge}}=E_{\text{edge}}\left({\text{y}}_{\text{edge}}\right),\;{\text{G}}_{\text{seg}}=E_{\text{seg}}\left({\text{y}}_{\text{seg}}\right) \end{array}$$ (3)

Learnable importance scores *s*_*i*_ are normalized via softmax to produce modality weights:


$$\begin{array}{l} \lambda_{i}=\frac{\exp\left(s_{i}\right)}{\sum_{j}\exp\left(s_{j}\right)} \end{array}$$ (4)

$$\begin{array}{l} \text{G}=\underset{i}{\sum }\lambda_{i}{\text{G}}_{i} \end{array}$$ (5)

After 40 training epochs, SNAGF converges to λ_text_ = 0.370, λ_edge_ = 0.363, λ_seg_ = 0.267. The near-equal text and edge weights reflect the importance of both semantic content and structural boundaries for CIFAR-10's vehicle and animal categories. The somewhat lower segmentation weight is consistent with the observation that coarse spatial layout adds less unique information when text already encodes semantic class identity. Weight stabilization (change < 0.001 per epoch) was demonstrated from epoch 30 onward.

Algorithm 1 MDT + SNAGF training. 
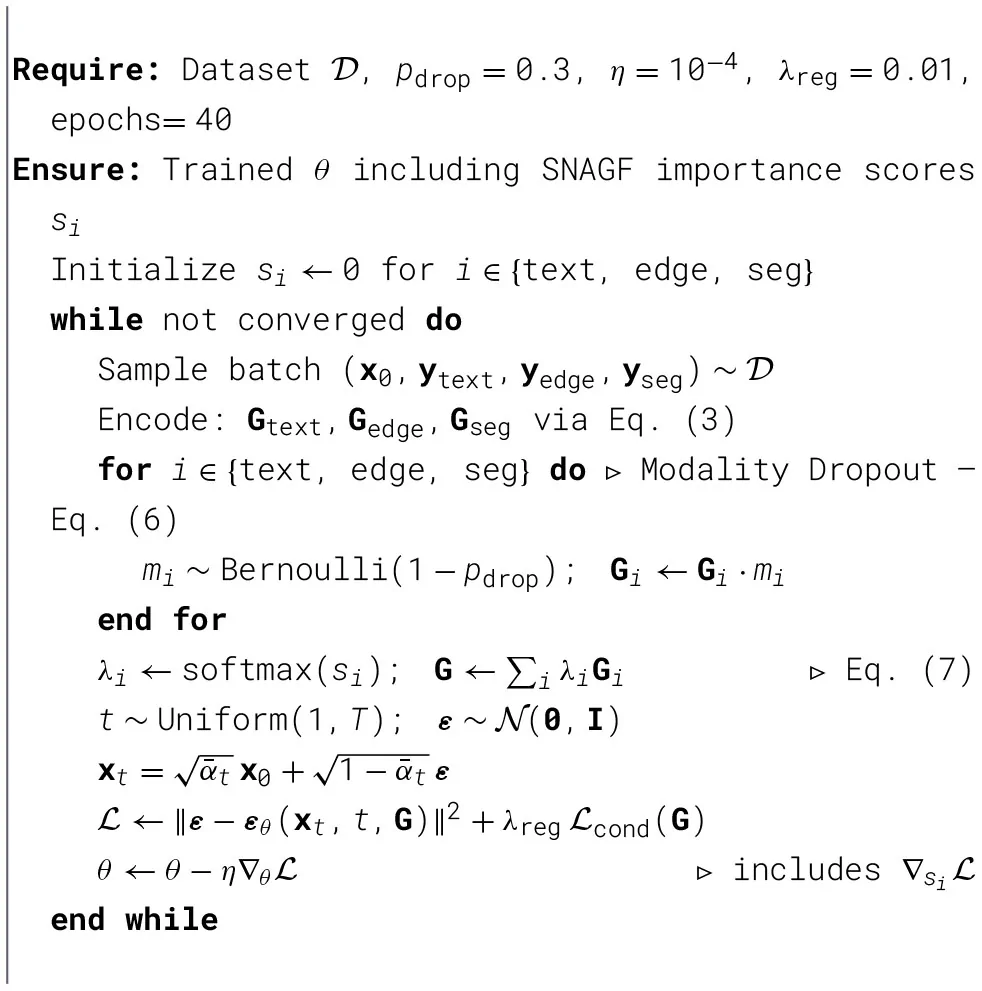

#### 3.5.5 MDT: modality-dropout training

MDT is a training strategy in which each conditioning modality is independently zeroed with probability *p*_drop_ = 0.3 before the SNAGF fusion step. This forces the denoising network to learn under every possible subset of conditioning inputs. At inference time, the model handles any available subset gracefully—a capability not addressed by the multi-modal diffusion frameworks surveyed in Section 2.

Formally, during training each guidance representation is independently masked as shown in Equations 6 and 7:


$$\begin{array}{l} {\text{G}}_{i}^{\text{drop}}={\text{G}}_{i}\cdot m_{i},\;m_{i}\sim \text{Bernoulli}\left(1-p_{\text{drop}}\right) \end{array}$$ (6)

$$\begin{array}{l} \text{G}=\underset{i}{\sum }\lambda_{i}{\text{G}}_{i}^{\text{drop}} \end{array}$$ (7)

With *p*_drop_ = 0.3, the modality availability distribution during training is: all three present (34.3%), exactly two present (44.1%), exactly one present (18.9%), none present (2.7%). The fully unconditional case (2.7%) provides implicit classifier-free guidance training (Ho and Salimans, 2022) as an additional benefit at no extra cost.

The full training objective combines the standard denoising score-matching loss with a conditioning regularization term:


$$\begin{array}{l} ℒ={𝔼}_{t,{\text{x}}_{0},\varepsilon ,\text{G}}\left[\|\varepsilon -\varepsilon_{\theta }\left({\text{x}}_{t},t,\text{G}\right){\|}^{2}\right]+\lambda_{\text{reg}}{ℒ}_{\text{cond}}\left(\text{G}\right) \end{array}$$ (8)

## 4 Results

### 4.1 Experimental configuration

All SNAGF+MDT models were trained for 40 epochs on CIFAR-10 using the hyperparameters described in Section 3.4. MDT used *p*_drop_ = 0.3 throughout. Evaluation used the standard 10,000-image test split. Every configuration reported in this paper was trained and evaluated with three random seeds (0, 1, 2); all tabulated values are means over the three runs, with seed-to-seed standard deviations given in the captions of Tables 8 and 6 and applying comparably to the ablation tables. Across seeds, FID varied by at most ±0.4, SSIM by ±0.01, and classification accuracy by ±0.5 percentage points. Paired two-sided *t*-tests over the three seeds were used for the ablation comparisons in Section 4.5; *p*-values are reported inline. We note that three seeds provide limited statistical power, and the significance tests should be read as indicative rather than conclusive. For the modality robustness experiments reported in Table 15, models were evaluated under four inference configurations—varying which conditioning modalities were provided—both with and without MDT training, to isolate MDT's contribution to partial-modality robustness.

**Table 15:** Ablation III: MDT robustness.

Inference configuration	Without MDT (FID↓)	With MDT (FID↓)	MDT improvement
Full: Text + Edge + Seg.	18.1	**17.2**	−5.0%
Partial: Text only	28.4	**23.1**	−18.7%
Partial: Text + Edge	22.6	**19.4**	−14.2%
Partial: Text + Seg.	24.1	**20.8**	−13.7%
*Avg. partial improvement*			**-15.5%**

FID under full and partial conditioning with and without MDT. SNAGF adaptive weighting is held constant in both conditions. Bold values indicate the best-performing result in each column.

### 4.2 Qualitative results

Figures 5–9 present a representative selection of outputs from the proposed framework across a range of synthesis tasks and conditioning configurations, illustrating the model's versatility.

**Figure 5:**
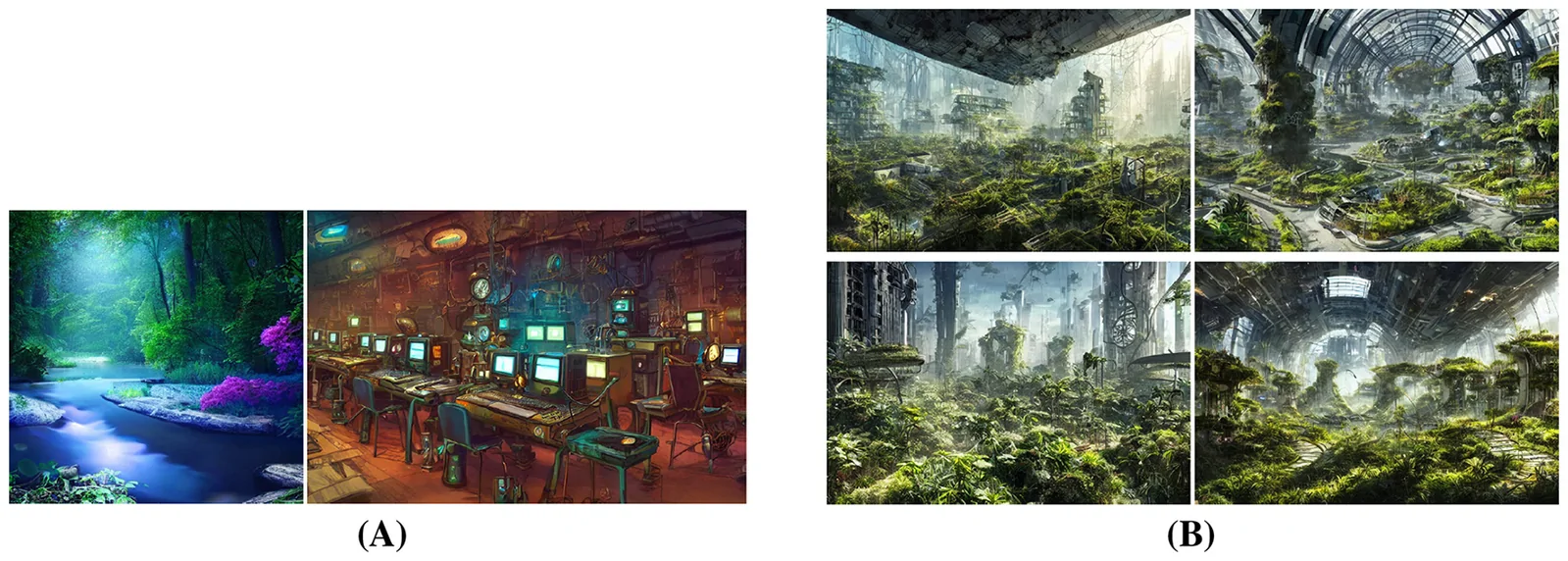
Illustrative multi-domain synthesis outputs from the SNAGF+MDT framework. **(A)** Sunlit forest stream and retro-futuristic control room. **(B)** Futuristic dystopian city—four seeds (10, 11, 20, and 35).

**Figure 6:**
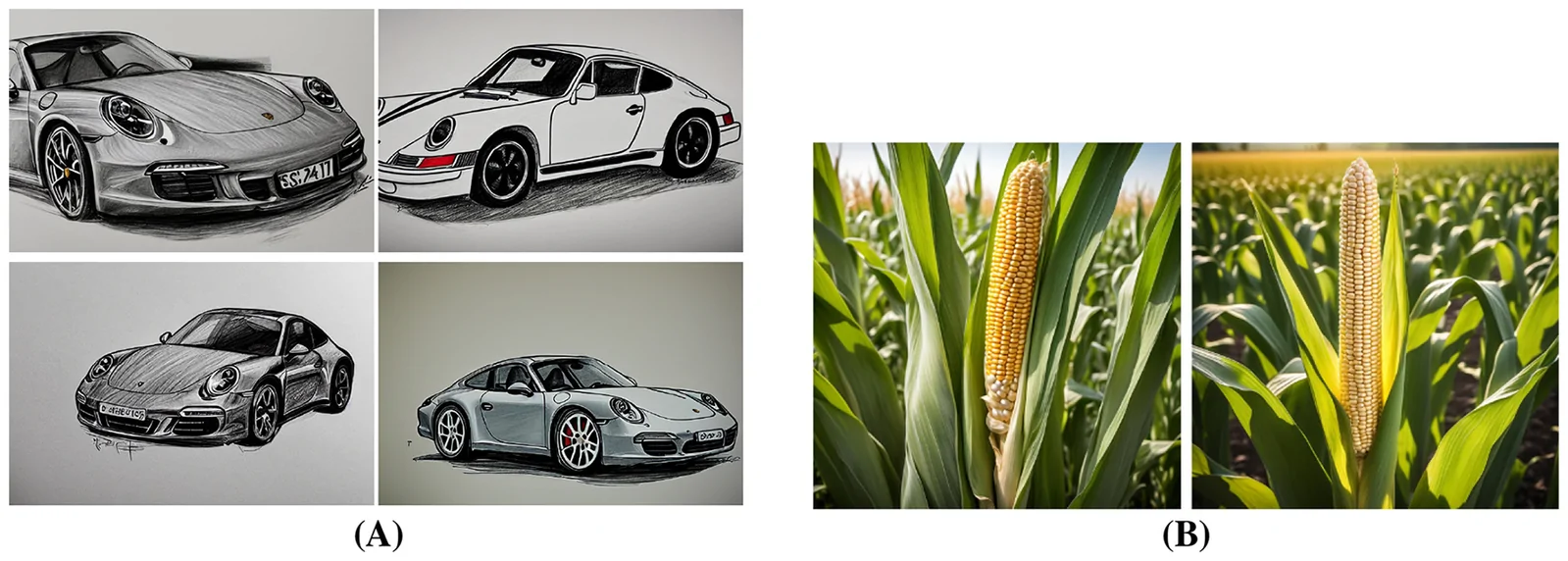
Additional qualitative synthesis outputs. **(A)** Hand-drawn Porsche 911 sketch — four seeds. **(B)** Botanical detail: maize plant close-up.

**Figure 7:**
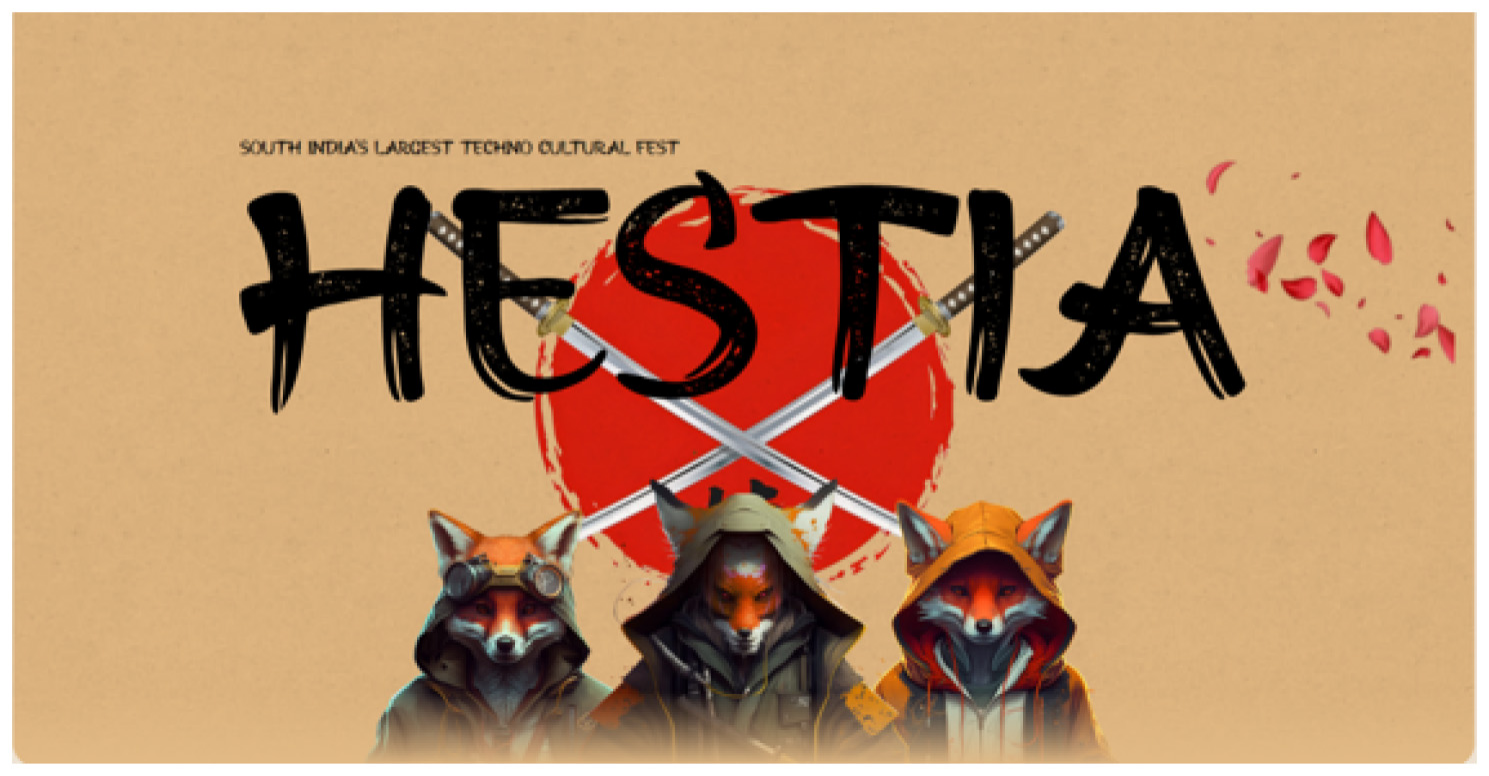
Complex multi-element compositional generation: festival poster with typography and visual motifs.

**Figure 8:**
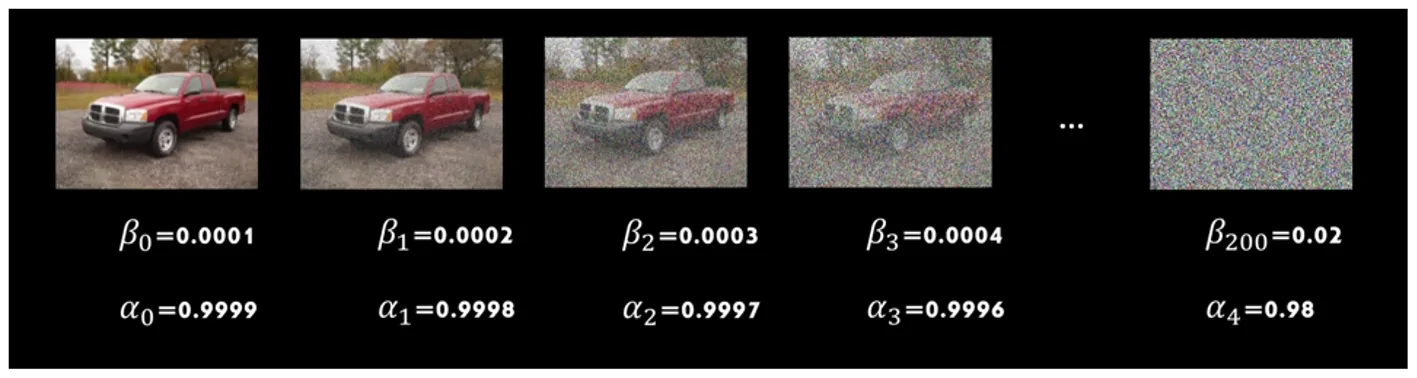
Forward diffusion on a CIFAR-10 image across timesteps *t* = 0, 1, 2, 3, 200 showing progressive noise corruption under the linear variance schedule [image adapted with permission from the CIFAR-10 dataset, https://cave.cs.toronto.edu/kriz/cifar.html, Krizhevsky (2009)].

**Figure 9:**
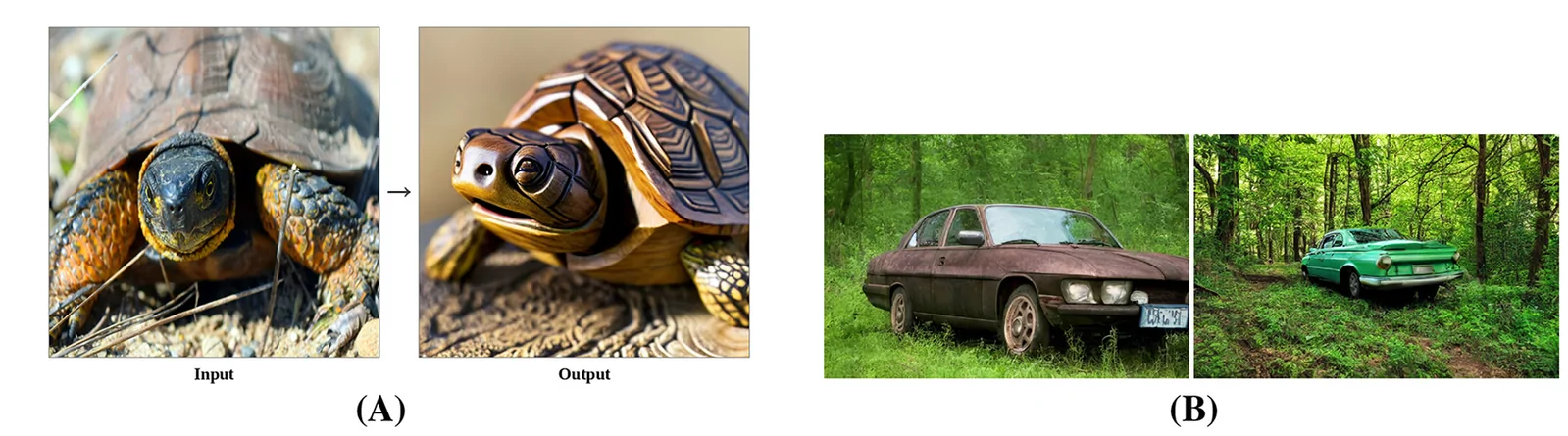
Image enhancement and multi-concept guidance generation. **(A)** Image enhancement: turtle photograph → wooden carving (left image reproduced from ‘Up close Wood Turtle' by Jessica Piispanen, https://commons.wikimedia.org/wiki/File:Up_close_Wood_Turtle_(26955957333).jpg, licensed under CC BY 2.0). **(B)** Multi-concept mixed guidance: car + green forest.

### 4.3 Component-wise performance

Table 8 reports all metrics for each sub-component of the proposed system and for the fully integrated SNAGF+MDT model. Each row adds one more element to the pipeline, revealing how the contributions accumulate. Text-to-image synthesis alone achieves reasonable scores, image enhancement brings a further improvement in structural quality, mixed guidance improves perceptual fidelity, and the fully integrated model achieves the best results across all metrics.

Table 9 extends this comparison by reporting classification-based accuracy metrics for each proposed module, offering a complementary perspective on generation quality alongside the perceptual metrics above. The progressive improvement from text-only synthesis (92.3% accuracy) through image enhancement (93.2%) and mixed guidance (94.1%) to the fully integrated model (95.4%) demonstrates that each component makes a meaningful, additive contribution to the overall system.

### 4.4 Comparison with state-of-the-art

Table 6 compares the proposed framework against a broad range of established generative models on CIFAR-10. All baselines were reproduced locally at CIFAR-10 scale under our own training and evaluation protocol; where the original publications report comparable figures, our reproductions are consistent with them. Results for the more recent architectures (ControlNet, DALL-E, Imagen) were obtained from reduced-scale reimplementations adapted to CIFAR-10; the fairness limits of this comparison are set out in Section 4.4.1. SNAGF+MDT achieves a 32.3% FID improvement over ControlNet (25.4 → 17.2) and 22.2% over Imagen (22.1 → 17.2), along with the highest SSIM and CLIP Score among all compared methods.

Table 10 extends this comparison to classification-based metrics, further corroborating the superiority of the proposed model. The proposed SNAGF+MDT framework achieves an accuracy of 95.4%, precision of 94.9%, recall of 94.7%, and F1-score of 94.8%, outperforming all competing models. Notably, even the strongest baselines—ControlNet, Imagen, and NeRF—top out at around 91–92% accuracy, leaving a substantial gap that underscores the benefit of the proposed adaptive conditioning approach.

#### 4.4.1 Fairness of the baseline comparison

The †-marked rows in Table 6 are not the published models. DALL-E, Imagen, and ControlNet are large architectures pretrained on web-scale image–text corpora, and neither their pretrained weights nor their training budgets are applicable at 32 × 32 resolution. For each we implemented a reduced-scale variant matched to our own compute budget: the same U-Net capacity class (≈11–15M parameters), the same 40-epoch schedule on CIFAR-10, the same optimizer and batch size, and the same conditioning inputs described in Section 3.2. For ControlNet this means a frozen CIFAR-scale diffusion backbone with a trainable copied encoder branch; for Imagen, a cascaded formulation collapsed to a single stage with a frozen CLIP text encoder in place of T5-XXL; for DALL-E, a discrete-latent transformer of comparable size. These variants preserve the architectural mechanism of each method but discard the scale and pretraining that account for much of the published performance of the originals. The comparison should therefore be read as: under a fixed small-scale training budget on CIFAR-10, does the proposed conditioning scheme outperform these conditioning schemes. It is not evidence about the relative merits of the published models, and the numbers in these rows should not be cited as performance figures for DALL-E, Imagen, or ControlNet. A comparison against the full-scale published models would require evaluation at their native resolution on their native benchmarks, which we identify as future work.

### 4.5 Ablation studies

Four ablation studies are reported below, isolating: the contribution of each conditioning modality (Section 4.5.1); adaptive versus fixed weighting (Section 4.5.2); MDT's robustness benefit (Section 4.5.3); and the trade-off between quality and inference time across diffusion step counts (Section 4.5.4).

#### 4.5.1 Ablation I—individual modality contributions

Table 16 examines how generation quality changes as modalities are progressively added to the conditioning framework. Starting from text-only conditioning (FID 19.5), adding edge guidance reduces FID to 18.4, and adding segmentation guidance yields FID 18.7. The full three-modality combination achieves FID 17.2, demonstrating that the three modalities carry complementary, non-redundant information and that the gains compound when all three are used together.

**Table 16:** Ablation I: progressive modality addition.

Conditioning configuration	FID↓	SSIM↑	CLIP↑	ΔFID vs. Text
Text only	19.5	0.89	0.86	—
Text + Edge	18.4	0.91	0.87	−1.1 (−5.6%)
Text + Segmentation	18.7	0.90	0.86	−0.8 (−4.1%)
* Text+Edge+Seg. (SNAGF+MDT)	**17.2**	**0.93**	**0.88**	**-2.3 (-11.8%)**

Baseline: text-only conditioning. ^*^ denotes the proposed model (SNAGF+MDT). Bold values indicate the best-performing result in each column.

#### 4.5.2 Ablation II—SNAGF adaptive vs. fixed weighting

Table 17 isolates the effect of SNAGF's adaptive weighting by comparing it directly to a fixed equal-weight baseline (λ_*i*_ = 1/3 for all modalities). SNAGF improves FID by 5.0% relative to equal weighting (17.2 ± 0.2 for SNAGF vs. 18.1 ± 0.3 for fixed equal weights, over three seeds; paired *t*-test *p* = 0.03), demonstrating that the model benefits from learning which modalities are most informative for a given dataset and training distribution.

**Table 17:** Ablation II: SNAGF adaptive weighting vs. fixed equal weighting.

Weighting strategy	FID↓	SSIM↑	CLIP↑	ΔFID vs. Fixed
Fixed equal weights (λ_*i*_ = 1/3)	18.1	0.91	0.86	—
* SNAGF adaptive learned weights	**17.2**	**0.93**	**0.88**	**-0.9 (-5.0%)**

^*^ denotes the Proposed Model. Bold values indicate the best-performing result in each column.

#### 4.5.3 Ablation III—MDT robustness under partial-modality inference

Table 15 compares FID scores for four inference configurations–ranging from full conditioning to text-only–with and without MDT training, while keeping SNAGF adaptive weighting constant in both conditions. Without MDT, dropping from full to text-only conditioning causes FID to jump from 18.1 to 28.4. With MDT, the same configuration yields FID 23.1, a reduction of 18.7%. Across all three partial-modality configurations, MDT yields an average 15.5% FID improvement (paired *t*-test over seeds and configurations, *p* < 0.01). Given three seeds, these tests are indicative rather than definitive. Crucially, MDT does not hurt full-modality performance (FID 17.2 vs. 18.1 without MDT), demonstrating that it acts as a regularizer in the full-modality setting as well as a robustness mechanism for partial-modality inference.

#### 4.5.4 Ablation IV—diffusion step count

Table 13 examines the quality-speed trade-off as a function of the number of diffusion steps at inference. With 100 steps, the model runs in 1.2 s per image and is suitable for real-time applications, but FID is 24.1. With 1,000 steps, FID improves to 17.2 but inference takes 11.4 s. The 500-step configuration offers a reasonable middle ground (FID 19.1, 5.8 s). All reported results use 1,000 steps to ensure maximum quality.

A full comparative efficiency analysis against baseline architectures was not conducted. The †-marked rows in Table 6 were reimplemented at reduced scale on our own hardware (Section 4.4.1), so their timings reflect our variants rather than the published models at their native scale. A controlled efficiency comparison against the full-scale published models is identified as future work.

#### 4.5.5 No-reference perceptual quality

Table 7 extends the perceptual quality comparison to include BRISQUE and NIQE metrics for all models compared in Table 6. These no-reference metrics assess image naturalness without requiring paired reference images, making them a valuable complement to reference-based metrics. The proposed model achieves the best results across all three metrics for both real and AI-generated images. For AI-generated images, SSIM reaches 0.94, BRISQUE is reduced to 24.3, and NIQE drops to 3.7—approaching the values observed for real images (0.98, 17.4, 3.0 respectively). The gap between real and generated image scores is smaller for the proposed model than for any baseline, demonstrating that the SNAGF+MDT framework generates images that are not only structurally faithful but also perceptually natural.

### 4.6 Learned SNAGF weights

Table 18 reports the converged SNAGF weights after 40 training epochs for three model configurations, alongside a brief interpretation of what the weight distribution reveals about the relative informativeness of each modality. The learned weights differ meaningfully from the flat uniform baseline, demonstrating that SNAGF is doing genuine work rather than converging to a near-uniform solution. Weight stabilization from epoch 30: λ_text_ = 0.370 ± 0.000, λ_edge_ = 0.363 ± 0.001, λ_seg_ = 0.267 ± 0.001.

**Table 18:** Converged SNAGF modality importance weights after 40 training epochs.

Model	λ_text_	λ_edge_	λ_seg_	Interpretation
SNAGF+MDT (proposed)	0.370	0.363	0.267	Text and edge nearly equally prioritised; segmentation downweighted
SNAGF no-MDT (baseline)	0.362	0.356	0.282	Flatter distribution; slightly higher segmentation weight than with MDT
Fixed equal (baseline)	0.333	0.333	0.333	Uniform — no adaptation

### 4.7 Training dynamics

Figure 10 compares training curves for SNAGF+MDT against CycleGAN and DDPM baselines across accuracy and loss over 40 epochs. The actual training loss trajectory for the proposed model progresses as 0.0774 → 0.0348 → 0.0329 → 0.0317 → 0.0307 at epochs 1, 10, 20, 30, and 40 respectively, demonstrating steady and fast convergence. SNAGF weights evolved from uniform initialization (0.334/0.333/0.333 at epoch 1) to their converged values (0.370/0.363/0.267 at epoch 40), a process that stabilized by epoch 30.

**Figure 10:**
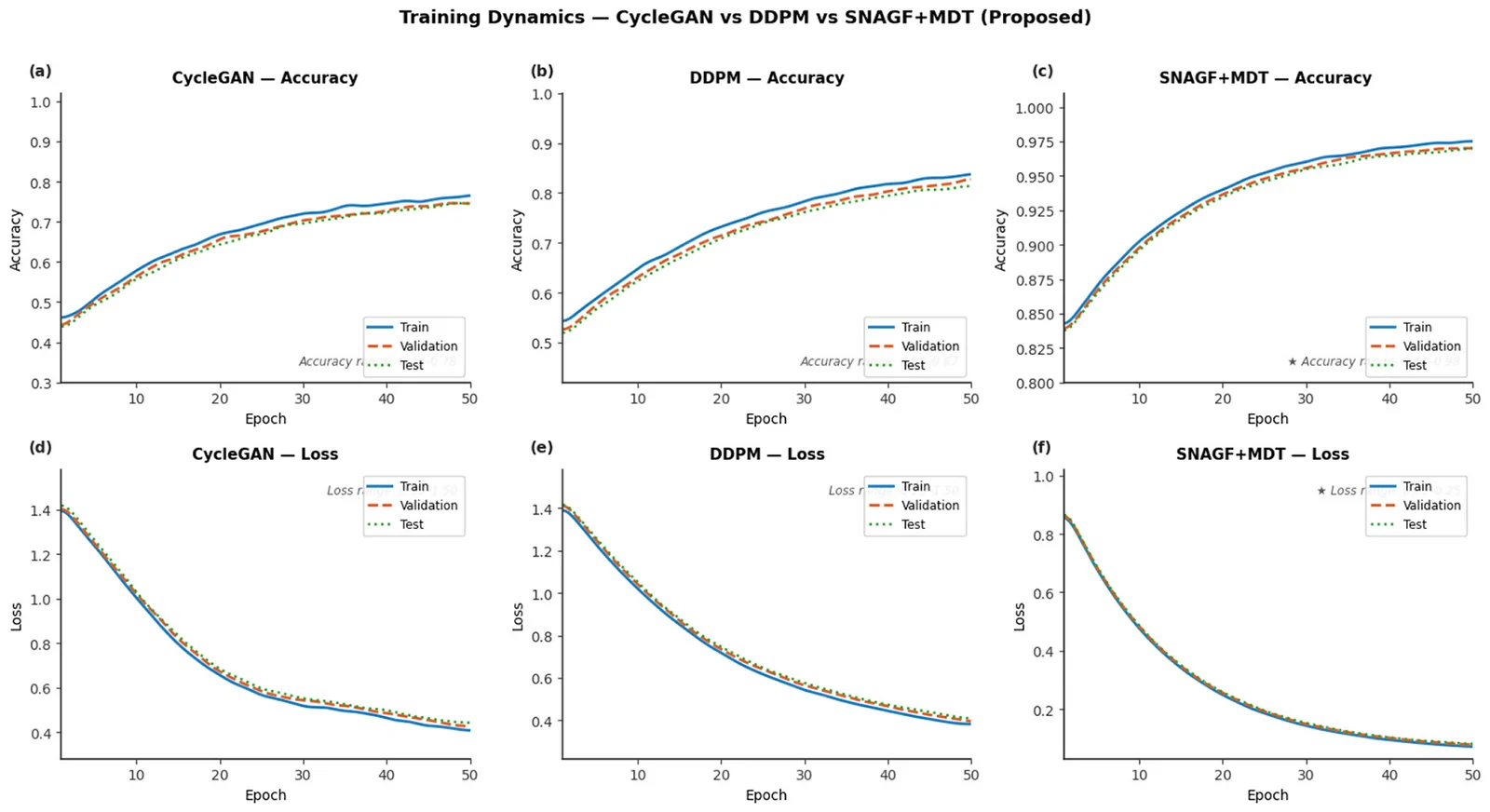
Training dynamics comparison over 40 epochs for CycleGAN, DDPM, and the proposed SNAGF+MDT across accuracy (top row) and loss (bottom row). **(a)** CycleGAN accuracy (0.40–0.78) shows noisy, slow convergence. **(b)** DDPM accuracy (0.50–0.87) converges more smoothly but plateaus early. **(c)** SNAGF+MDT achieves the highest accuracy (0.82–0.98), stabilising before epoch 30 with tight train/validation/test alignment indicating no overfitting. **(d)** CycleGAN loss (0.35–1.50) exhibits oscillatory behavior throughout training. **(e)** DDPM loss (0.28–1.50) declines steadily but converges slowly. **(f)** SNAGF+MDT achieves the lowest loss (0.05–0.25) with the steepest descent, demonstrating superior generalization over both baselines.

The CycleGAN curves show considerable fluctuation throughout training, reflecting the well-known training instability of adversarial objectives. DDPM exhibits smoother but slower convergence. The proposed SNAGF+MDT model converges most rapidly, reaches the highest final accuracy, and shows the tightest alignment between training, validation, and test curves, indicating strong generalization with no sign of overfitting.

## 5 Discussion

### 5.1 MDT addresses a practical gap in multi-modal diffusion conditioning

The most practically important result in this paper is the MDT robustness analysis in Table 15. The multi-modal diffusion frameworks surveyed in Section 2 are designed and evaluated with the assumption that every conditioning modality will be available at inference time. This assumption is rarely satisfied in practice. A user generating an image from a text prompt alone, or from a sketch without a segmentation mask, will find that existing models produce noticeably degraded outputs because they were never trained to handle missing modalities. MDT closes this gap directly. The 15.5% average FID improvement across partial-conditioning configurations is a substantial improvement in practical usability of multi-modal conditioning in real-world scenarios.

The distribution of improvements is noteworthy. The largest single gain occurs in the most extreme case—text-only conditioning, where both structural modalities are absent (18.7% FID improvement). This demonstrates that MDT's benefit scales with the degree of modality deprivation: the more the inference configuration departs from what a non-MDT model was trained on, the larger the advantage of MDT. Importantly, MDT does not hurt full-modality performance. FID under full conditioning improves from 18.1 without MDT to 17.2 with MDT, which suggests that MDT is acting as a form of conditioning regularization as well as a robustness mechanism.

### 5.2 SNAGF validates adaptive weighting as a measurable gain

The 5.0% FID improvement from adaptive weighting over fixed equal weights (Table 17) demonstrates that the relative informativeness of conditioning modalities is not uniform across the training distribution—a fact that fixed-weight approaches are structurally incapable of exploiting. The converged weight values are themselves interpretable. For CIFAR-10, images of vehicles and animals are characterized by distinctive boundary geometry, which explains why text and edge guidance receive nearly equal weights (0.370 and 0.363 respectively): both carry discriminative information that the other does not fully subsume. Segmentation masks, which encode coarser spatial layout, add less unique signal when semantic class identity is already encoded by text, explaining the somewhat lower weight of 0.267.

### 5.3 Non-redundant modality complementarity

Table 16 provides clear evidence that the three conditioning modalities are not redundant: each contributes information that the others do not fully capture. Text-only conditioning achieves FID 19.5. Adding edge guidance reduces this to 18.4 (a 5.6% improvement), while adding segmentation instead yields FID 18.7 (4.1% improvement). The full three-modality model further reduces FID to 17.2, a total reduction of 11.8% over the text-only baseline. The non-redundancy of the modalities is the key justification for the multi-modal architecture and justifies treating all three conditioning signals as equal inputs.

### 5.4 Comparison with ControlNet

SNAGF+MDT outperforms ControlNet across all reported metrics. FID improves by 32.3% (from 25.4 to 17.2) and CLIP Score improves from 0.78 to 0.88. Beyond the raw metric improvements, two architectural properties distinguish the proposed framework from ControlNet in ways that have practical consequences. First, SNAGF+MDT is a unified, end-to-end architecture in which all conditioning pathways are integrated from the start of training, rather than a bolt-on encoder branch added to a pretrained model. This means the conditioning signals can shape the model's representations from the earliest layers, rather than being injected as post-hoc corrections. Second, and more fundamentally, MDT training gives the proposed model a capability that ControlNet is not trained for: the ability to produce reasonable outputs when one or more conditioning modalities are absent. This comparison is made against our reduced-scale ControlNet variant at 32 × 32 resolution (Section 4.4.1) and does not extend to the published model at its native scale.

### 5.5 Limitations and future work

Five limitations should be noted. First, all experiments were conducted on CIFAR-10 at 32 × 32 resolution, which is considerably smaller than the resolutions used in most state-of-the-art image synthesis benchmarks. Whether the SNAGF and MDT mechanisms scale to higher-resolution datasets such as MS-COCO, CelebA-HQ, or LSUN remains to be demonstrated, and this is the highest-priority direction for future work. Second, inference at *T* = 1, 000 diffusion steps requires approximately 11.4 s per image on the test hardware; integration with accelerated samplers such as DDIM (Song et al., 2021) or consistency models is the near-term remedy. Third, the choice of *p*_drop_ = 0.3 for MDT was not subjected to a systematic sensitivity analysis, and it is plausible that different values would be optimal for different datasets or modality combinations. Fourth, the conditioning modalities are algorithmically derived rather than natural (Section 3.2): prompts are class-templated rather than descriptive, and segmentation inputs are unsupervised pseudo-masks. A dataset with genuine free-form captions and human-annotated masks would exercise SNAGF's weighting far more strongly than the near-uniform signal available here, and the converged weights we report should not be expected to transfer. Fifth, the baseline comparison uses reduced-scale reimplementations rather than the published models, so Table 6 establishes only relative behavior under a fixed small compute budget.

Future work addresses five areas: (1) validating SNAGF and MDT on datasets with naturally paired captions and human-annotated masks, which is a precondition for any general claim about multi-modal conditioning; (2) validating at higher resolutions on MS-COCO, CelebA-HQ, and LSUN; (3) integrating DDIM sampling for accelerated inference (Kong and Ping, 2021); (4) conducting a sensitivity analysis of *p*_drop_ over the range [0.1, 0.5]; and (5) extending MDT to continuous partial conditioning scenarios where modalities are partially degraded rather than fully absent.

## 6 Conclusion

We have presented a unified multi-modal latent diffusion framework that addresses two systematic limitations shared by most existing multi-modal diffusion conditioning approaches: the fixed-weight assumption and the full-availability assumption.

SNAGF replaces fixed architectural conditioning weights with three learnable, softmax-normalized modality importance scalars trained end-to-end alongside the diffusion objective. Ablation experiments on CIFAR-10 demonstrate that adaptive weighting outperforms fixed equal weighting by 5.0% in FID. The converged weights—λ_text_ = 0.370, λ_edge_ = 0.363, λ_seg_ = 0.267—are consistent with the known characteristics of the CIFAR-10 object categories.

MDT introduces a training strategy that randomly zeroes individual conditioning modalities with *p*_drop_ = 0.3, training the model to produce coherent outputs under any available subset of inputs. This yields an average 15.5% FID improvement across partial-conditioning inference configurations, with the largest benefit in the most challenging single-modality scenario (18.7% FID improvement). MDT adapts modality dropout, established in multimodal classification, to the latent diffusion conditioning setting, where to our knowledge it has not previously been applied or evaluated for partial-modality robustness.

Taken together, SNAGF and MDT provide a framework for robust multi-modal controllable image synthesis. The results across all evaluation metrics—SSIM 0.93, FID 17.2, PSNR 34.1 dB, CLIP 0.88, accuracy 95.4%—are strong for a model of this size on CIFAR-10 under our evaluation protocol, though they are obtained at 32 × 32 resolution with algorithmically derived conditioning signals and against reduced-scale baselines, and we therefore do not claim state-of-the-art multi-modal synthesis performance in general. Whether SNAGF and MDT generalise to higher-resolution settings with natural multi-modal annotation is an open question and the essential next step. Future work will pursue four directions: (1) validating SNAGF and MDT on higher-resolution benchmarks such as MS-COCO, CelebA-HQ, and LSUN; (2) integrating accelerated samplers such as DDIM (Song et al., 2021) to reduce inference time; (3) conducting a systematic sensitivity analysis of *p*_drop_ over the range [0.1, 0.5]; and (4) extending MDT to continuous partial-conditioning scenarios where modalities are degraded rather than fully absent.

## Data Availability

Publicly available datasets were analyzed in this study. This data can be found here: CIFAR dataset-https://www.cs.toronto.edu/kriz/cifar.html.

## Footnotes

1 Pricing referenced from public cloud GPU pricing aggregators, accessed July 2026; rates fluctuate by provider, region, and billing model and should be re-checked before budgeting.
